# Electrophoretic Patterns of *Toxoplasma* *gondii* Excreted/Secreted Antigens and Their Role in Induction of the Humoral Immune Response

**DOI:** 10.5812/jjm.9525

**Published:** 2014-04-01

**Authors:** Ahmad Daryani, Mehdi Sharif, Hamed Kalani, Alireza Rafiei, Farzad Kalani, Ehsan Ahmadpour

**Affiliations:** 1 Toxoplasmosis Research Center, Mazandaran University of Medical Sciences, Sari, IR Iran; 2 Molecular and Cell Biology Research Center, Mazandaran University of Medical Sciences, Sari, IR Iran

**Keywords:** Immunization, *Toxoplasma*, Immunoblotting

## Abstract

**Background:**

*Toxoplasma gondii* is an obligatory intracellular protozoan parasite on which studies are pending regarding production of vaccine. To date, the production of human vaccine has not been successful where approximately one third of the world’s population is thought to be infected with *T. gondii*.

**Objectives:**

The present study was designed to compare the electrophoretic patterns of *T. gondii* excreted/secreted antigens (ESAs) and determine their role in the stimulation of the humoral immune response.

**Materials and Methods:**

*T. gondii* ESAs were prepared from cell cultures (albino rat fibroblast) and cell-free mediums (RPMI-1640). Next, the SDS-PAGE technique was used for comparing molecular weights of the antigens. Forty C57BL/6 mice were divided randomly into four groups (n = 10). Immunization was performed subcutaneously at an interval of 2 weeks in two groups by injecting 100 µg of each of the above-mentioned antigens. Two groups, as negative control, also received fibroblast lysate proteins or adjuvant separately. All of the groups were then challenged with the *T. gondii* RH strain. Serum samples were collected from all mice and measured by immunoblotting technique for detection of immunogenic antigens.

**Results:**

The electrophoretic mobility of the prepared antigens/proteins from cell culture, cell-free media, Fibroblast Lysate Proteins and *Toxoplasma *Lysate Antigens (TLA) showed 13, 12, 8 and 8 bands, respectively. The case groups, in challenge with *T. gondii* (RH strain), showed more survival prolongation than the control groups. Furthermore, the survival period was identical for both case groups with a tendency for slightly higher survival of mice receiving ESA from cell-free medium. Analysis of sera by immunoblotting also revealed one band of 65 KDa in sera from both case groups.

**Conclusions:**

We suggest that this band be extracted and its amino acids sequence determined to produce Synthetic Polypeptide for immunization studies.

## 1. Background

*Toxoplasma gondii* is an obligatory intracellular protozoan parasite that infects a wide range of warm-blooded vertebrates as intermediate hosts and felines as definitive hosts. Although it is benign in normal individuals, toxoplasmosis can be complicated in infants and people with the Acquired Immune Deficiency Syndrome (HIV positive/AIDS), undergoing chemotherapy, and recent organ-transplant recipients. Moreover, most infants have no symptoms at birth, but a small percentage may be born with eye or brain damage because of this parasite (1, 2). Children born with toxoplasmosis can be afflicted with mental retardation, convulsions, spasticity, cerebral palsy, deafness, microcephalus, and hydrocephalus. 

The most common finding in congenital toxoplasmosis is retinochoroidit is that can lead to blindness (2-4). *T. gondii* can also cause repeated attacks of encephalitis in people with HIV Positive/AIDS or a number of other immune system disorders that may result in death or long-term neurological defects (2). Inevitably, the singular or at least the best way of controlling the disease is the use of an effective vaccine for the definitive host (cats) or intermediate hosts. Because of the large population of cats, it is not possible to access all such felines simultaneously and also due to the lack of an effective vaccine against toxoplasmosis in cats, there is no choice but to try to focus on developing a vaccine to prevent toxoplasmosis in intermediate hosts. So far, success in the field of vaccine production has only been for sheep. 

A live attenuated vaccine (Toxovax) has been used in livestock for some time and has been successful in limiting abortion in sheep (1, 5). To date, the production of human vaccine has not been successful despite the fact that about one third of the world’s population is thought to be infected with *T. gondii*, and the number of people at risk is increasing progressively. Hence, it is indispensable to produce an effective human vaccine against *T. gondii*. Immunization studies with virulent live parasites (6-8), killed parasites (9, 10), radiation-attenuated strains (11, 12), or parasitic antigens (13-16) showed only partial protection against *T. gondii* leading to research for other protective antigens. It has been demonstrated that the ESAs of *T. gondii *tachyzoites are highly immunogenic and thus might be good candidates for vaccine development against *T. gondii *(2).

## 2. Objectives

Contrary to other studies, in this research, we prepared *T. gondii* excreted/secreted antigens without using FBS and compared the electrophoretic patterns of the different types of antigens and also analyzed the role of these antigens in the stimulation of the humoral immune response. As well, we evaluated the survival rate of immunized mice with these antigens responding to the challenge with wild strain of *T. gondii* tachyzoites (RH strain).

## 3. Materials and Methods

### 3.1. Parasites

The RH strain of *T. gondii *tachyzoites were harvested from lavage of the peritoneal cavity of Swiss-Webster mice injected 4 days previously with 0.5 mL of parasite suspension in sterile cold Phosphate-Buffered Saline (PBS; pH = 7.4). Each sample obtained from individual mice was divided into two equal parts, centrifuged at 100 ×g for 10 minutes at 4˚C to remove peritoneal cells and debris. The supernatant was again centrifuged at 200 ×g for 10 minutes at 4˚C to obtain the free *Toxoplasma *tachyzoites. The parasite pellets were washed twice in PBS, containing 100 IU/mL penicillin and 100 µg/mL streptomycin, and the number of parasites were determined by counting in a hemacytometer under phase-contrast microscopy (400X). The yield of parasites derived from the above procedure was 7-13 × 10^7^mL, of which 97% excluded trypan blue dye (4, 14, 17).

### 3.2. Mice

Female 8 to 10-week-old C57BL/6 inbred mice were used for immunization experiments. The project underwent ethical review and was given approval by the Ethics Committee of Mazandaran University of Medical Sciences. Care and use of experimental animals complied with local animal welfare laws, guidelines and policies.

### 3.3. Isolation and Culture of Fibroblast

For isolation of fibroblast cells albino rats weighing 80-120 g were used. A piece of skin measuring approximately 4 cm^2^ from the belly region of an anesthetized rat was cut with sterile scissors and forceps, washed several times in RPMI-1640 medium supplemented with 10% FBS, penicillin (100 U/mL) and streptomycin (100 mg/mL)-complete medium- and then cut into small pieces of about 2 mm^2^. Four small pieces were cultured in a 25 cm^2^ flask with 10 mL complete medium (see above), incubated in a humidified incubator at 37˚C, under 5% CO_2_ in air. After two to five days, the fibroblasts adhered, migrated, proliferated and became confluent (18, 19).

### 3.4. Preparation of Antigens/Proteins

#### 3.4.1. Fibroblast Proteins

The adherent fibroblasts were washed three times with PBS in order to remove FBS. The cells were scraped off the surface of the flask with a plastic cell scraper which was previously cooled in a -20˚C freezer. The cell suspension was transferred into a centrifuge tube with 1 mL of cold distilled water. Immediately, 5 mM phenylmethylsulphonyl fluoride (PMSF)-as a protease inhibitor- was added to it to prevent proteolytic activity. The solution was freeze-thawed three times at -80˚C and +37˚C and then centrifuged at 12000 ×g in a precooled centrifuge for 15 minutes. The supernatant was harvested and transferred to a fresh centrifuge tube and the pellet was discarded. The sample was divided into aliquots and its protein concentration was determined using the Bradford method and stored at -20˚C until used.

#### 3.4.2. *Toxoplasma Lysate* Antigens

Approximately 25 × 10^6^ live tachyzoites of the RH strain of *T. gondii* were used for preparation of TLA. For this, the tachyzoites were washed twice in cold PBS by centrifuging at 750 ×g for 10 minutes at 4˚C with brakes on and the supernatant was discarded. The pellet was re-suspended by adding 1 mL of distilled water and then supplemented with 5 mM PMSF. The suspension was freeze-thawed 3 times at -80˚C and +37˚C and its protein concentration was determined by the Bradford method and subsequently kept frozen at -20˚C until used (20).

### 3.5. Excretory/Secretory Antigens

#### 3.5.1. ESA Obtained From Cell Culture

Tachyzoites were added to 25 cm^2^ tissue culture flasks with confluent fibroblasts (1:1) which were previously washed three times with PBS to remove FBS. Forty-eight hours later, the culture supernatants were harvested, pooled and supplemented with 100 IU/mL penicillin, 100 µg/mL streptomycin and 5 mM PMSF and then passed through a 0.22 µm Millipore membrane filter and its protein concentration was measured by the Bradford Method and stored at -20˚C until used (19).

#### 3.5.2. ESA Obtained From Cell-free Medium

Under sterile conditions, tachyzoites were obtained by centrifuging peritoneal fluids of infected mice and then washed three times with RPMI-1640 medium. Afterwards, approximately 5-8 × 10^6^mL tachyzoites were re-suspended in a sterile 15 mL conical centrifuge tube in 2 mL of the same medium plus 100 IU/mL penicillin and 100 µg/mL streptomycin. The tubes were incubated horizontally at 37˚C for 3 hours under mild pulsation and then centrifuged at 1000 ×g for 10 minutes and supernatants were collected, pooled and filtered by passing through a 0.22 µm Millipore membrane filter and its protein concentration was determined using the Bradford method and stored at -20˚C until used (12).

### 3.6. Electrophoresis by SDS-PAGE Method

The antigens/proteins of *T. gondii *tachyzoites were separated on a 10% sodium dodecyl sulfate-polyacrylamide gel electrophoresis (SDS-PAGE) as described by Laemmli, 1970. Briefly, the samples including antigens/proteins from cell culture, cell-free medium, fibroblast lysate proteins and TLA were prepared by boiling in the Laemmli sample buffer in a 95-100˚C water bath for five minutes and loaded on top of the gel with 4% (w/v) acrylamide stacking gel and 10% (w/v) acrylamide resolving gel. SDS-PAGE was carried out in Tris-glycine buffer (pH = 8.3), using a constant current of 100 V for 1 hour. After electrophoresis was completed, the gel was stained with Coomassie brilliant blue R-250 to visualize the protein bands on the gel. The relative molecular mass of each recognized band was determined by comparison with standard molecular markers (21).

### 3.7. Immunization and Challenge

Forty female C57BL/6 mice, 8-10 weeks of age, were randomly divided into four groups (n = 10). Two groups were used as control and two groups as case. The control groups received 200 µL Freund’s Complete Adjuvant (FCA) or 200 µg Fibroblast Lysate Proteins. The case groups were also immunized separately with ESAs derived from cell culture or cell-free media. Immunization was done subcutaneously in two doses at an interval of 2 weeks by injecting 100 µg of each above-mentioned antigen plus 100 µL FCA. After one week, all groups were challenged subcutaneously with 2000 live tachyzoites to evaluate their survival period (14).

### 3.8. Immunoblotting

Immunoblotting was performed only for three samples including antigens/proteins from cell culture, cell-free medium and Fibroblast Lysate Protiens by the Towbin et al. (1979) Technique(22). Proteins separated by gel electrophoresis were transferred to a nitrocellulose membrane at 100 V in a Tris-glycine transfer buffer for one hour. After Ponceau S staining, the membrane was cut into 4-5 mm wide strips and then blocked in 5% nonfat dry milk in Tris-Buffered Saline with 0.05% Tween 20 (TBS-T) at room temperature for 30 minutes. Then, the strips were washed three times in TBS-T, incubated with serum samples at room temperature for 1 hour and washed again with TBS-T to remove unbound antibodies. The strips were then incubated with Horseradish Peroxidase (HRP)-conjugated goat anti-mouse IgG antibody diluted 1:500 in TBS-T at room temperature in the dark for 1 hour. After final washing, the strips were soaked in substrate solution consisting of 0.5 mg/mL Diaminobenzidine (DAB) and 0.1% Hydrogen Peroxide in TBS (pH = 7.5) to visualize immunoreactive bands.

### 3.9. Statistical Analysis

The survival function was estimated according to a match given by Kaplan and Meier. The life-table method required that the observations be grouped into 24-hour intervals. The Wilcoxon Test was utilized for measuring the differences between survival curves. In this test, the null hypothesis presumes that there is no real difference between survival curves for the three compared groups.

## 4. Results

### 4.1. SDS-PAGE

**Figure 1.**
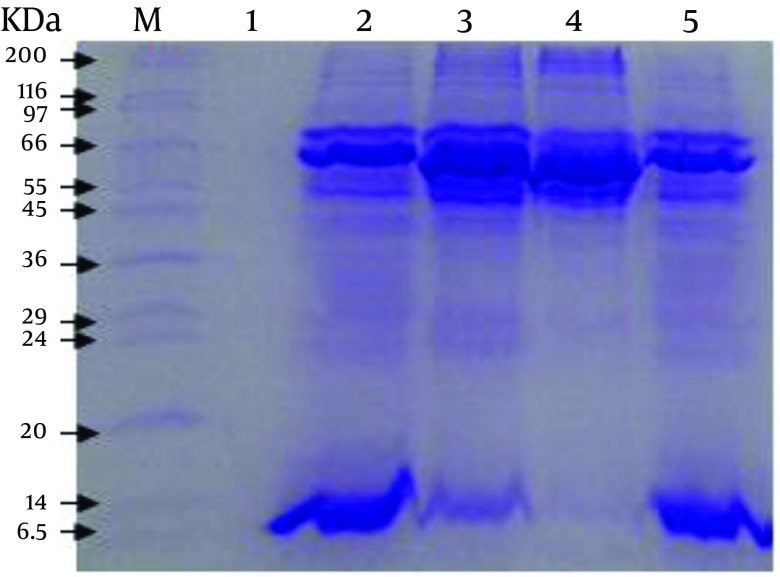
Comparison of Different Patterns of *Toxoplasma* Proteins by SDS-PAGE M, marker, wide range 6.5 to 200 KDa; 1, supernatant from third washing of tachyzoites; 2, ESA from cell free medium; 3, ESA from cell culture; 4, extract of fibroblast; 5, Toxoplasma Lysate Antigens (TLA).

The SDS-PAGE of antigens/proteins from the cell culture, cell-free medium, TLA and Fibroblast Lysate Proteins revealed 13 (13.5, 23.5, 25, 39, 42, 48.5, 54, 65, 75, 154, 191, 200 and 220.5 KDa), 12 (13.5, 23.5, 25.5, 36.5, 39, 40.5, 50.5, 52, 65, 75, 154 and 191 KDa), 8 (13.5, 39, 40.5, 48.5, 65, 75, 154 and 165.5 KDa) and 8 (42, 48.5, 54, 65, 75, 154, 200 and 220.5 KDa) polypeptide bands, respectively (Figure 1). 

### 4.2. Immunization and Challenge

The results of the challenge experiments in mice immunized with cell culture ESA, cell free medium ESA and adjuvant are presented in Table 1. Two control groups including mice that received only adjuvant and those that received Fibroblast Lysate Proteins died within 6 and 7 days, respectively when challenged subcutaneously with 2000 tachyzoites of RH strain. In comparison with the control groups, mice of the two case groups which received separate ESAs from cell culture or cell-free media died within 9 days. Significant protection of mice immunized with different kinds of ESA has been observed in contrast to a lack of protection in mice immunized with adjuvant (P < 0.05).

**Table 1.** Cumulative Mortality Frequency of Different Groups of Mice Immunized by Various Antigens ^a,^ ^b^

Days After Infection	Cumulative Mortality Frequency Percent in Mice Groups Injected With			
	CC-ESA	CF-ESA	FLP	Adjuant
**4**	0	0	0	0
**5**	0	0	30	37.5
**6**	37.5	11.2	60	100
**7**	62.5	55.6	100	-
**8**	75	66.7	-	-
**9**	100	100	-	-

^a^ Abbreviations: CC-ESA, Cell Culture ESA; CF-ESA, Cell Free Medium ESA; FLP, Fibroblast Lysate Proteins.

^b^ Groups of 10 mice received two injections of immunogen pre challenge with 2000 RH strain tachyzoites. Results plotted as number of mice surviving per day for the five groups.

### 4.3. Immunoblotting

**Figure 2.**
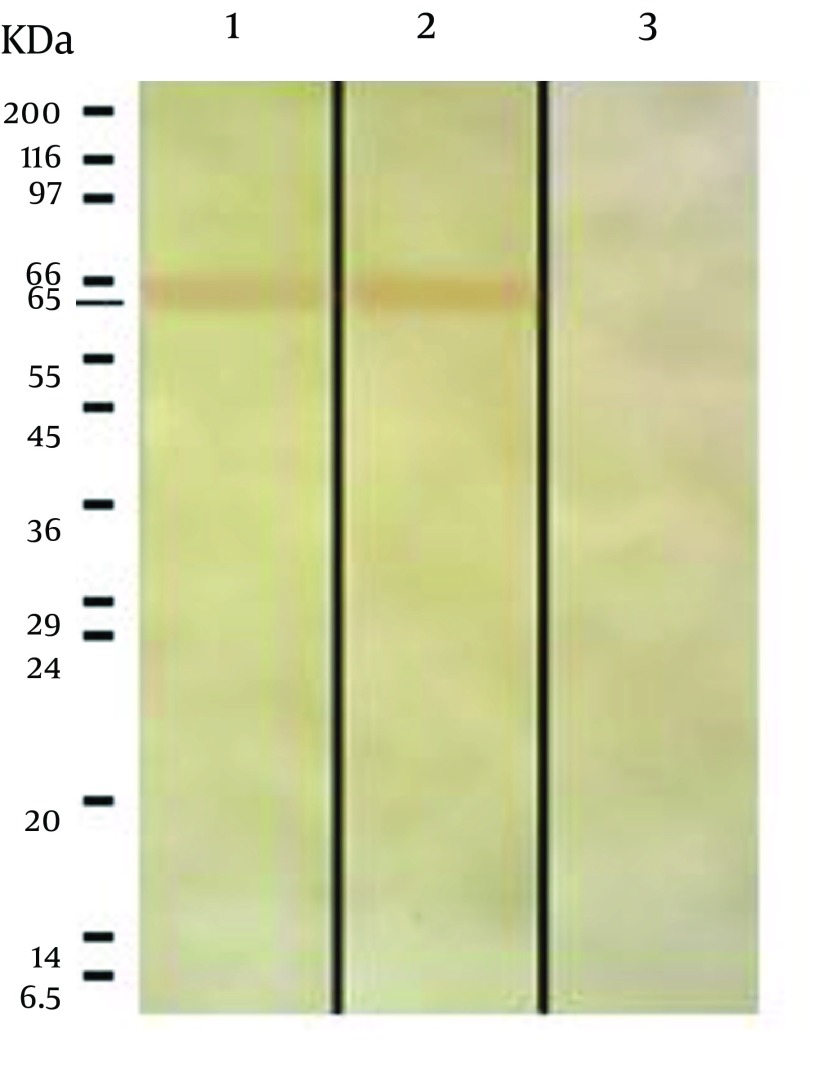
Immunoblotting Result for Preparations of *T. gondii* 1, ESA from cell free medium; 2, ESA from cell culture; 3, Extract of fibroblast; A band of 65 KDa reacted with serum of immunized mice.

The immunoblotting showed one band of 65 KDa in both ESAs obtained from cell culture and from cell-free medium. No reaction was observed in the Fibroblast Lysate sample, which was used as a negative control (Figure 2). 

## 5. Discussion

During studies of different types of *T. gondii* antigens, it has been demonstrated that ESAs can induce stronger protection than other types of antigens, and this is one reason why researchers have paid so much attention to ESAs. Although ESAs are strongly immunogenic in mice and induce a humoral immune response to prevent spread of the disease, studies on the survival of mice challenged with different strains of *T. gondii*, particularly RH strain, showed that immunized mice with ESAs would die after a few days compared to the control group (s). This implies that the parasite can perhaps deviate immune responses towards a Th2 pattern by antigen shedding, so that the antibodies produced in response to the antigens (ESAs) may be not very effective on intracellular parasites. 

Three current methods for preparation of *T. gondii* ESAs are from cell-free media (14), cell culture (16), and peritoneal exudate of infected mice (23). As use of serum (including FBS) in cell culture media is an essential factor for growth and proliferation of cells and even after washing the cells several times with PBS traces of albumin may be observed in the contents of total ESAs by SDS-PAGE Technique (16); additionally, the existence of serum proteins in ESAs can bring about incorrect estimation of ESAs contents. Some proteins may also be present in ESAs derived from peritoneal exudate of infected mice as a result of the secretion of peritoneal cells (no parasite) or cell lysis by the parasite (24). This is clear by what was mentioned above about ESAs influence on immunization. Therefore, it seems that ESAs harvested from cell-free media, without adding serum into the medium, have the highest purity in comparison with other types of ESAs; because only *T. gondii *tachyzoites are present in the medium and clearly ESAs obtained by this method are the yield of secretion and excretion of the parasites. However, in the present study we found that there are only some differences in the electrophoretic mobility of the prepared antigens/proteins (Figure 1). 

Darcy et al. reported that 1.8 × 10^8^ parasites are requirement for producing 12 to 20 micrograms of ESAs from cell-free medium (24). In our study, however, approximately 12.5 mg of ESAs was prepared from about 25 × 10^9^ live tachyzoites, which was four times higher than what Darcy et al. reported (24). High-speed centrifuge (approximately 1000×g or above) can disrupt tachyzoites and also decline their viability. Therefore, we utilized low-speed centrifuge to harvest a greater number of viable tachyzoites for preparation of more ESA. The results of SDS-PAGE showed that molecular weights of antigen/protein bands for samples obtained from cell-free medium, cell culture, Fibroblast Lysate Protein, and TLA are in range from 13.5 to 191, 13.5 to 220.5, 42 to 220.5 and 13.5 to 165.5 KDa, respectively. 

Three common bands were observed in all the mentioned samples with the molecular weights of 154, 75 and 65 KDa. However, the nature of the bands are different from each other because the 65 KDa band relevant to Fibroblast Lysate Proteins showed no reaction with mice serum while the 65 KDa bands in both ESAs from cell-free medium and cell culture reacted with mice serum (Figure 2). Furthermore, three bands with molecular weights of 42, 48.5 and 54 KDa were observed in both ESA sample from cell culture and in the sample of Fibroblast Lysate Proteins. Since the 42 and 54 KDa bands were not observed in ESA sample from cell-free medium, it may be relevant to fibroblast proteins. However, *T. gondii* has a 54 KDa (ROP2) antigen where reduction in synthesis and secretion of the antigen can decrease the parasite virulence (25, 26), and of course, another protein with the molecular weight of 42 KDa (ROP6) has proteolytic activity and plays an important role in active invasion by *T. gondii *(27), where its presence in ESA from cell culture and its absence in ESA from cell-free medium indicates that this band may be ROP6, which is secreted by *T. gondii* during the active invasion process into fibroblast cells. Hence, the 42 KDa band in ESA from cell culture differs from that in Fibroblast Lysate Proteins in terms of amino acids sequence and structure. 

The other common bands in electrophoresis of ESA from cell culture and Fibroblast Lysate Proteins are 200 and 220.5 KDa bands, and it seems that these are related to fibroblast cells because no antigens with molecular weight of more than 200 KDa in relation to *T. gondii* ESAs have been reported (24). It is also important that *T. gondii *tachyzoites can secrete several antigens under various conditions (28-30). Daryani et al. in their study reported some bands with molecular weights of 20.1, 33, 44, 47, 52, 55, 58, 66-69, 86, 110, 135, 150, 165 and 175 KDa in samples of total ESA from RPMI-1640 without adding FBS (14). In the current study, only two bands of 65 KDa and 52 KDa were observed among above antigens. The 52 KDa antigen may be related to ROP8, which is secreted by the parasite into the host cell cytoplasm and mitochondrial membrane during and even after invasion (25, 31), but the 65 KDa antigen is not seen among the list of *T. gondii* ESAs. Nevertheless, the antigen could be expected to be a 66 KDa antigen known as ROP2 that is secreted by rhoptries and acts as an important factor in the pathogenesis of *T. gondii *(25, 26).

Son and Narm (2001) reported 15 bands with molecular weights of 110, 97, 86, 80, 70, 60, 54, 42, 40, 36, 30, 28, 26, 22 and 19 KDa in sample of ESAs from Hank’s Balanced Salt Solution (HBSS) (32). Among above antigens the 40, 36 and 26 KDa antigens are most likely the same as the 40.5, 36.5 and 25.5 KDa antigens in our study. It appears that the above-mentioned 40 KDa antigen is GRA4 (33) and the 36 KDa antigen may also be GRA10 or ROP9 (31, 34).The results of immunization also showed that the mice of the two control groups receiving either adjuvant alone or Fibroblast Lysate Proteins died within six or seven days after being challenged with *T. gondii* (RH strain). The case groups showed more survival prolongation than the control group, and the survival time was identical for both test groups with a tendency for slightly higher survival period for mice receiving ESA from cell-free medium. 

Costa-Silva et al. in their study used 1000 tachyzoites of RH strain of *T. gondii* for the challenge experiment, and immunized mice with ESAs died after three days with respect to the control group, namely the survival period difference between immunized and control groups of mice was three days (16). Daryani et al. showed that the survival rate of mice in the group receiving ESA-F2 was much higher than the control group, the main reason being due to purification of ESAs using Ion Exchange Chromatography (14). 

The result of immunoblotting revealed only one band with molecular weight of 65 KDa in both samples of ESAs, which reacted with immunized mice serum. Although the 65 KDa antigen detected by immunoblotting can lead to a strong humoral immune response, it seems not to be very effective on intracellular parasites, so that one mechanism by which the parasite evades host immune system is through antigen shedding. Meanwhile, the 65 KDa band has been rarely reported in other studies. The 65 KDa band reacted with antibodies of immunized mice serum, so we suggest that this band be extracted and its amino acids sequence for producing synthetic polypeptide determined. This can also be used for further studies on immunization against *T. gondii*.
